# Relationship of Epstein-Barr Virus and Interleukin 10 Promoter Polymorphisms with the Risk and Clinical Outcome of Childhood Burkitt Lymphoma

**DOI:** 10.1371/journal.pone.0046005

**Published:** 2012-09-27

**Authors:** Carolina Minnicelli, Mário H. M. Barros, Claudete E. Klumb, Sérgio O. Romano, Ilana R. Zalcberg, Rocio Hassan

**Affiliations:** 1 Bone Marrow Transplantation Center (CEMO), Instituto Nacional de Câncer (INCA), Rio de Janeiro, Brazil; 2 Hematology Service, Instituto Nacional de Câncer (INCA), Rio de Janeiro, Brazil; 3 Pathology Department (DIPAT), Instituto Nacional de Câncer (INCA), Rio de Janeiro, Brazil; University of Nebraska - Lincoln, United States of America

## Abstract

Epstein-Barr virus (EBV) is an important environmental factor associated to the development of Burkitt lymphoma (BL) in endemic and intermediate risk regions. However, little is known about the contribution of genetic constitution to the development and clinical response of the disease. The aim of this work was to investigate the role of EBV and Interleukin 10 (*IL10*) single nucleotide polymorphisms (−1082A/G, −819C/T, −592C/A) and microsatellites (IL10.R and IL10.G) in susceptibility and clinical outcome in pediatric BL patients, in a region with intermediate EBV association frequency. The frequencies of *IL10* promoter Single nucleotide polymorphisms −1082A/G, −819C/T, −592C/A, and IL10.R and IL10.G microsatellites were compared in 62 pediatric patients and 216 healthy donors. *IL10* −1082GG and GCC/GCC genotypes were more frequent in patients than in controls, and associated to a higher risk of BL development (GG genotype OR 2.62, 95% CI, 1.25–5.51; *P* = 0.008; *Pc* = 0.024). EBV was detected in tumor samples by EBER-ISH in 54.1% of cases. EBV+ patients exhibited a better event free survival (EFS) (*P* = 0.019) than EBV− patients. Carriers of *IL10* R3-GCC had worse EFS (*P* = 0.028). Our results suggest a risk effect and an independent prognostic value of *IL10* polymorphisms and EBV in childhood BL patients.

## Introduction

Burkitt lymphoma (BL) is an aggressive non-Hodgkin lymphoma (NHL) of mature B cells which varies in respect to age-specific incidence, primary site of tumor and Epstein-Barr virus (EBV) association in different geographic situations [1], [2]. These differences allowed for the characterization of the endemic (eBL) and sporadic (sBL) subtypes; the former affecting young African children, with involvement of abdomen and facial bones, and the latter occurring worldwide, mainly affecting young adults, with involvement of the terminal ileum and lymph nodes [1], [2]. Respective to infection status, eBL is almost always EBV-associated while sBL shows a more irregular association, ranging from 10% to 30% EBV+ cases in different geographical areas [1], [3]. In Brazil, BL is mainly a childhood disease, and EBV association ranges from 80% in Northeast [4] to 50–60% in the Southeast region of the country [5]–[7]. We have previously proposed an intermediate risk status for childhood BL arisen in South-East Brazil [5], [8], where a causal relation between age of EBV seroconversion and BL risk was suggested. However, the exact role of EBV in BL pathogenesis or disease progression has been elusive until now [9].

*MYC* oncogene deregulation resulting from its translocation to one of the immunoglobulin genes is the common pathogenic event for all BL subtypes, which can account for most of the cellular disease features [10]. However, the causes of BL epidemiological and clinical variability are hidden by the complexity of environmental interactions that might act in different geographical situations where the tumor arises. Host factors influencing BL genetic risks are largely unknown, which prompted us to investigate the potential involvement of interleukin 10 (*IL10*) gene polymorphisms in disease susceptibility and clinical response.

*IL10* is part of an immunoregulatory cytokine network, produced mainly by monocytes, macrophages, T cells, and normal and neoplastic B cells [11]. Besides the immunosuppressor effects mediated by this cytokine, which may support an environment favorable to neoplastic cells expansion [12], *IL10* can also exert proliferative effects on B cells, particularly in BL cells, acting as a paracrine or autocrine proliferative factor [13].

Familiar and twin studies showed that 50–75% of inter-individual variability in *IL10* levels can be attributed to genetic variations [14], [15]. Three Single Nucleotide Polymorphisms (SNPs), −1082(A/G), −819(C/T) and −592(C/A), localized in the proximal gene promoter, exert a functional effect on *IL10* expression [16]. These polymorphic sites are in linkage disequilibrium (LD) and form haplotype families when combined to microssatellites IL10.G (−1.2 Kb) and IL10.R (−4 Kb) [17].

Being IL10 an indispensable B-cell survival factor, it is not surprising that EBV has developed mechanisms to coopt its biological properties. EBV is capable of inducing human IL10 in B cells through its viral RNAs [18], [19]. Moreover, it encodes a viral *IL10* (vIL-10), which is ∼80% homologue and shares many of the biological properties of the human cytokine [11]. vIL10 has important roles in inducing B cell growth and tolerizing the immune responses during pre-latent phase or lytic reactivaction [11], [20]. Reinforcing the role of this cytokine in EBV infection, human *IL10* polymorphisms were associated to the timing and susceptibility of primary EBV infection in a healthy Finland population [21], [22]. In the light of the demonstrated biological interactions between *IL10*, BL and EBV infection, a case-control study was performed to investigate the role of *IL10* gene promoter polymorphisms in BL pathogenesis, as well as in the therapeutic response, in a region with intermediate EBV association frequency.

## Methods

### Ethics Statement

This study was approved by the Instituto Nacional de Câncer (INCA) Ethics Committee, and has therefore been performed in accordance with the ethical standards of the Declaration of Helsinki and the Brazilian rule 196/96 of the Conselho Nacional de Saúde. All patients and controls were included in the study after written informed consent. Guardians have signed the informed consent on the behalf of the children participants involved in this study.

### Patients and Pathological Samples

Sixty-two children (up to 16 years old), diagnosed with Burkitt lymphoma/leukemia at the Instituto Nacional de Câncer (INCA), Rio de Janeiro, Brazil, were included in this study, based on availability of pathology specimens and clinical records.

Histopathological diagnosis was based on morphological criteria and immunohistochemical detection of CD20 and CD10 and a high proliferation rate (Ki-67 almost 100%), according to the WHO classification [2]. Samples consisted of fresh biopsies and/or bone marrow cells in 50 cases, and formalin-fixed paraffin embedded tissues (FFPET) in the remaining twelve. Disease staging was performed according to the St. Jude's/Murphy staging classification for childhood Non-Hodgkin lymphoma [23].

Intensity of treatment was adjusted to risk groups (RG). Patients in risk group 1 (RG1) presented with St Jude's stage I or II, and one completely resectable lymphomatous lesion. RG2 patients presented with non-resectable extra-abdominal primary disease or stage II abdominal tumor with lactic acid dehydrogenase (LDH) augmented less than two times the normal levels.

High risk disease (risk group 3, RG3) included patients with abdominal tumor (St. Jude stage III), LDH up to 1000 U/l, involvement of BM or central nervous system or multimodal bone disease (St. Jude stage IV), as well as patients classified as L3 acute lymphoblastic leukemia [24]. All children were treated according to the NHL-Berlin-Frankfurt-Munster (BFM) protocols backbone strategies, and among them, 39 were uniformly treated with the LNH-98 INCA institutional protocol [24].

### Controls

The control group included 63 children (1–16 years; median 11) and 153 adults (17–61 years; median 36), totalizing 216 healthy individuals from the same geographic region (Rio de Janeiro State), registered for bone marrow or peripheral blood donation at Bone Marrow Transplantation Center (CEMO – INCA), a public institution offering hematopoietic stem cell transplantation to the National Health System patients in Rio de Janeiro, Brazil. Sex ratio between male and female was 2.2∶1.

Donors belonged to a comparable socio-economic and ethnic origin as BL patients, since they were selected from the relatives of transplant candidates at our hospital in the same period of the present study, excluding lymphoma patient relatives. Ethnic matching of patients and controls was not taken into account in the experimental design since relevant studies showed that skin color or self referred ethnic origin are not good markers of ancestry in Brazil [25]. Moreover, previous comparisons of *IL10* gene polymorphisms between normal Afro- and Euro-descendents persons from the same geographical region (Rio de Janeiro State) and African and European populations did not disclose any ethnical stratification in Brazilians [26].

### EBV Detection

EBV was detected by RNA in situ hybridization, performed on FFPET sections using fluorescein isothiocyanate (FITC)-conjugated EBV-encoded RNAs (EBERs) oligonucleotides probes (EBER-ISH). Detection of hybridized sites was achieved using a monoclonal antibody anti-FITC labeled with alkaline phosphatase (Novocastra Laboratories Ltd., Newcastle upon Tyne, UK).

### Sample Preparation

High molecular weight DNA was obtained from fresh samples (biopsies, bone marrow or peripheral mononuclear cells) with standard procedures. FFPET-DNA was extracted from 5 µm-sections, following strict measures for avoiding cross-contamination between samples, as described [27].

### PCR Assays

*IL10* SNPs −1082A/G and −592C/A were detected by Allele-specific (AS-) PCR, as described in Supplementary Table 1. SNP −592 (rs1800872) genotyping results were independently confirmed by using a TaqMan® allelic discrimination pre-developed assay (C_1747363_10) (Applied Biosystems, Foster City, CA, US), in an ABI PRISM 7700 Sequence Detector (Applied Biosystem), to confirm results obtained with conventional PCR methods.

**Table 1:** Clinical and demographic characteristics of Burkitt lymphoma patients.

Variable (N)	Category	Frequency (%)
Sex (62)	Masculine	43 (69.4)
	Feminine	19 (30.6)
Age group (62)	2–4 years	26 (41.9)
	5–9 years	28 (45.2)
	10–14 years	8 (12.9)
St. Jude's Stage^1^ (54)	I	6 (11.1)
	II	10 (18.5)
	III	24 (44.4)
	IV	14 (25.9)
Risk Group^2^ (54)	1	3 (5.6)
	2	19 (35.2)
	3	32 (59.3)

N: number of cases with available data.

1 St. Jude's staging according to Murphy (ref. 23).

2 Risk group as classified in the Berlin-Frankfurt-Munster (BFM) 90 protocol, adopted by the Instituto Nacional de Câncer – INCA LNH-98 (Brazil) protocol for the treatment of childhood Non Hodgkin lymphoma (ref. 24).

Based on the strong linkage disequilibrium between SNPs −819 and −592, three crossing AS-PCRs were simultaneously carried out using combinations of primers at positions −1082A/G, −819C/T and −592C/A, to experimentally confirm the haplotypes ACC, ATA and GCC (Table S1).

Alleles of microssatellites IL10.R and IL10.G (CA)_n_ were amplified with fluorescence labeled oligonucleotides, as previously described [28] and PCR fragments were analyzed in an automatic sequencer (MegaBACE 1000 DNA Sequencer; Amersham Pharmacia Biotech, Piscataway, NJ, US) with the Gene Profiler software.

All PCR reactions were carried out with final volumes of 30 µl containing 0.2 mM of each dNTP, 0.2 µM of each primer and 0.5 U of *Platinum Taq DNA Polymerase*, (Invitrogen, Carlsbad, CA, USA), from 50 ng of genomic DNA.

Positive and negative controls and 140 replicate samples were interspersed in the genotyping assays. Agreement for quality control replicates and duplicates was more than 98% for all assays.

### Statistical Analyses

Allele frequencies were calculated by the allele counting method. Hardy-Weinberg equilibrium was tested for each SNP by comparing observed with expected frequencies using a χ^2^ test. Three different genetic models were tested, including dominant model (comparing homozygous wild-type genotype with variant allele carrying genotypes), recessive (comparing wild-type allele-carrying genotypes with homozygous variant genotype), and additive model (Cochran-Armitage test). The best-fitting among the three models was the one with the smallest *p* value. The program R (R Development Core Team, 2006, http://cran.r-project.org) was used for genetic modeling, using a specific script developed for the purpose of the case-control analyses. Associations between polymorphisms and pathologic condition were tested using Fisher's exact and Pearson's χ^2^ tests. Magnitude of associations was calculated as *odds ratios* (OR) with 95% confidence interval (CI) by unconditional logistic regression analyses.

Event free survival (EFS) was the interval (in months) from diagnosis to progression at any time, relapse from complete response, or initiation of new, previously unplanned treatment or to the last follow-up in the patients with treatment success. EFS probabilities were estimated by the Kaplan-Meier method and compared by log-rank test. The multivariate Cox proportional hazard regression method was used to determine the independent prognostic value of statistically significant variables in univariate analyses, adjusted by sex and age when necessary. Data were analyzed using Statistical Package for the Social Sciences 13.0 (SPSS) and *P* values<0.05 were considered statistically significant.

## Results

### Patients' Characteristics

The patient's group was characterized by a predominance of male (M43∶ F19, sex ratio 2.26) and very young children (median age 5 years, 2–14). Complete clinical information was available for 54 children, with 38 (70.3%) being in advanced disease stages (III/IV) and 32 (59.3%) in RG3. Demographic and clinical data of patients are shown in Table 1.

### EBV Infection

EBV was detected in 33 of 61 (54.1%) cases tested. A relationship between lower age and EBV infection was observed (*P* = 0.008, Fisher's exact test).

### Genotype and Allele Frequencies of IL10 Promoter

Comparisons between adults and children in the control group revealed no difference regarding genotype/haplotype frequencies (Table S2); therefore, the entire control group was used in the case-control analysis.

A comparison of the genotype and allele frequencies of *IL10* −1082A/G and −592C/A polymorphisms between cases and controls is shown in Table 2. All genotype frequencies were in Hardy-Weinberg equilibrium in both groups (BL χ^2^ 1.13, *P* = 0.28; Controls, χ^2^ 0.03; *P* = 0.85 for −1082A/G and BL χ^2^ 0.016, *P* = 0.89; Controls, χ^2^ 0.004; *P* = 0.94 for −592C/A). The −1082GG genotype was overrepresented in patients (23% vs 10.2%), associated to a higher risk of BL development (OR 2.62, 95% CI, 1.25–5.51; *P* = 0.008). Significance of the genotype imbalance held true after Bonferroni correction for multiple comparisons (two polymorphisms and 3 genetic models, *Pc* = 0.024) In agreement, the haplotype combination GCC/GCC was also associated with BL risk (OR 2.53, CI 95% 1.2–5.32, *P* = 0.012; Pc = 0.072). No differences between cases and controls were found for −592C/A allelic or genotypic frequencies (Table 2).

**Table 2:** Case-control comparisons of genotypic and allelic frequencies of SNPs −1082 e −592 and genetic models of disease risk.

IL10 polymorphisms	BL^1^ (%)	Controls^1^ (%)	OR (95%CI)	*P* 2
**−1082A/G**	61 (100)	230 (100)		*0.021*
A/A	21 (34.4)	102 (47.2)	1.0 (reference)	-
G/A	26 (42.6)	92 (42.6)	1.37 (0.72–2.6)	0.2
G/G	14 (23)	22 (10.2)	3.09 (1.36–7.00)	0.0052
Recessive Model				*0.009*
AA/AG	47 (77)	194 (89.8)	1.0 (reference)	-
GG	14 (23)	22 (10.2)	2.62 (1.25–5.51)	0.008
Cochran – Armitage Test				0.0105
**−592C/A**	61 (100)	205 (100)		*0.302*
C/C	33 (54.1)	90 (43.9)	1.0 (reference)	-
C/A	24 (39.3)	92 (44.9)	0.71 (0.39–1.29)	0.26
A/A	4 (6.6)	23 (11.2)	0.473 (0.15–1.47)	0.189
Recessive Model				*0.67*
CC/CA	57 (93.4)	182 (88.8)	1.0 (reference)	-
AA	4 (6.6)	23 (11.2)	0.55 (0.18–1.67)	0.344
Cochran – Armitage Test				0.122

1 Numbers represent the number of individuals carrying each IL10 proximal genotype;

2 *P* values column: for genotype comparisons, the first p value (italics) represents the comparison of BL cases vs. controls, by Chi square test; and the following represent the significance of the odds ratio (OR), as calculated by logistic regression, having case vs. control as dependent variable and each IL10 genotype as a covariate.

**Figure 1:**
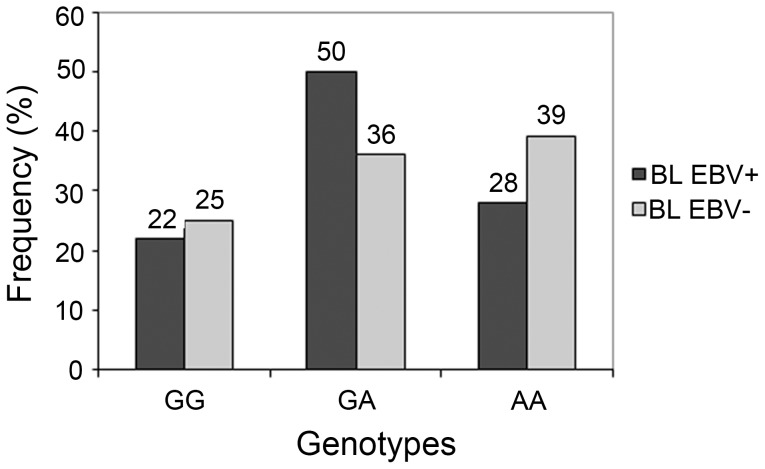
Genotypic frequencies of SNP-1082G/A in EBV+ and EBV− Burkitt lymphoma cases.

No statistical differences were observed in *IL10* polymorphisms frequencies between EBV+ and EBV− subgroups (Fig. 1).

### IL10 Microsatellites

IL10.G and IL10.R were analyzed in 462 chromosomes (102 BL and 360 controls). IL10.G alleles varied between G6 and G14; (CA)_18_ to (CA)_26_. Microssatellite IL-10.R alleles ranged from R1 to R4; (CA)_8,_ to (CA)_11_. No differences were observed between patients and controls for IL10.G and IL10.R alleles (*P* = 0.47 and *P* = 0.54, respectively, Monte Carlo simulation with 1000 bootstraps, CI 99%).

### IL10 Haplotype Families

*IL10* haplotype families were built according to Eskdale et al, 1999 [17], as follows: family IL10.01: IL10.R3-G-C-C; IL10.02: R2-A-C-C; IL10.03: R2-G-C-C; and IL10.04: R2-A-T-A. Three rare families were recorded both in patients and controls (Table 3). No statistical differences were observed in the frequencies of *IL10* families between patients and controls (102 and 360 chromosomes, respectively) (*P* = 0.165).

**Table 3:** Comparisons of haplotypes and combinations between BL patients and controls.

Haplotypes	BL^1^ (%)	Controls^1^ (%)	*P* 2	OR (95%CI)
**−1082/−819/−592**	**2N = 120**	**2N = 410**	*0.03*	
A C C	36 (30)	143 (34.9)	0.07	0.58 (0.32–1.04)
A T A	31 (25.8)	138 (33.6)	0.305	0.73 (0.41–1.3)
G C C	53 (44.2)	129 (31.46)	0.142	1.58 (0.87–2.86)
**Combinations**	N = 60	N = 205	*0.117*	
ACC/ACC	6 (10)	17 (8.3)	0.613	1.22 (0.46–3.27)
ATA/ATA	4 (6.7)	23 (11.2)	0.343	0.53 (0.17–1.61)
GCC/GCC	14 (23.3)	22 (10.7)	0.012	2.53 (1.2–5.32)
ACC/ATA	11 (18.3)	58 (28.3)	0.135	0.56 (0.27–1.17)
ACC/GCC	13 (21.7)	51 (24.9)	0.732	0.83 (0.41–1.66)
ATA/GCC	12 (20)	34 (16.6)	0.563	1.25 (0.6–2.61)
**Haplotype Families**	2N = 98/102	2N = 347/360	*0.165*	
IL10.01 (R3-GCC)	12 (11.8)	45 (12.5)	0.91	1.04 (0.5–2.17)
IL10.02 (R2-ACC)	33 (32.4)	122 (33.9)	0.406	0.768 (0.41–1.43)
IL10.03 (R2-GCC)	29 (28.5)	68 (18.9)	0.141	1.65 (0.88–3.09)
IL10.04 (R2-ATA)	24 (23.5)	112 (31.1)	0.083	0.565 (0.3–1.06)
**Rare Families**	2N = 4/102	2N = 13/360		
IL10.05 (R3-ATA)	1 (0.9)	5 (1.4)	-	-
IL10.06 (R3-ACC)	0 (0)	2 (0.6)	-	-
IL10.07 (R4-GCC)	3 (2.9)	6 (1.6)	-	-

1 N represent the number of individuals and 2N the number of chromosomes carrying each IL10 proximal haplotype or combination;

2 P values column: for each category, the first p value (italics) represents the comparison of cases vs. controls, by Chi-square Test; and the following represent the significance of the odds ratio (OR), as calculated by logistic regression, having case vs. control as dependent variable and each IL10 haplotype, combination or family as a covariate.

### Survival of BL Patients

Thirty nine patients received the same treatment and supportive care [24] and these were evaluated for OS and EFS, with a follow-up period ranging from 1 to 190 months (median 82 months). The EFS of the whole group was 79.5%. Risk group, despite not statistically significant, was the clinical characteristic that best distinguished patients with good and bad outcome (RG2 92.9% vs. RG3 69.6%, *P* = 0.11; Log-rank test).

**Figure 2:**
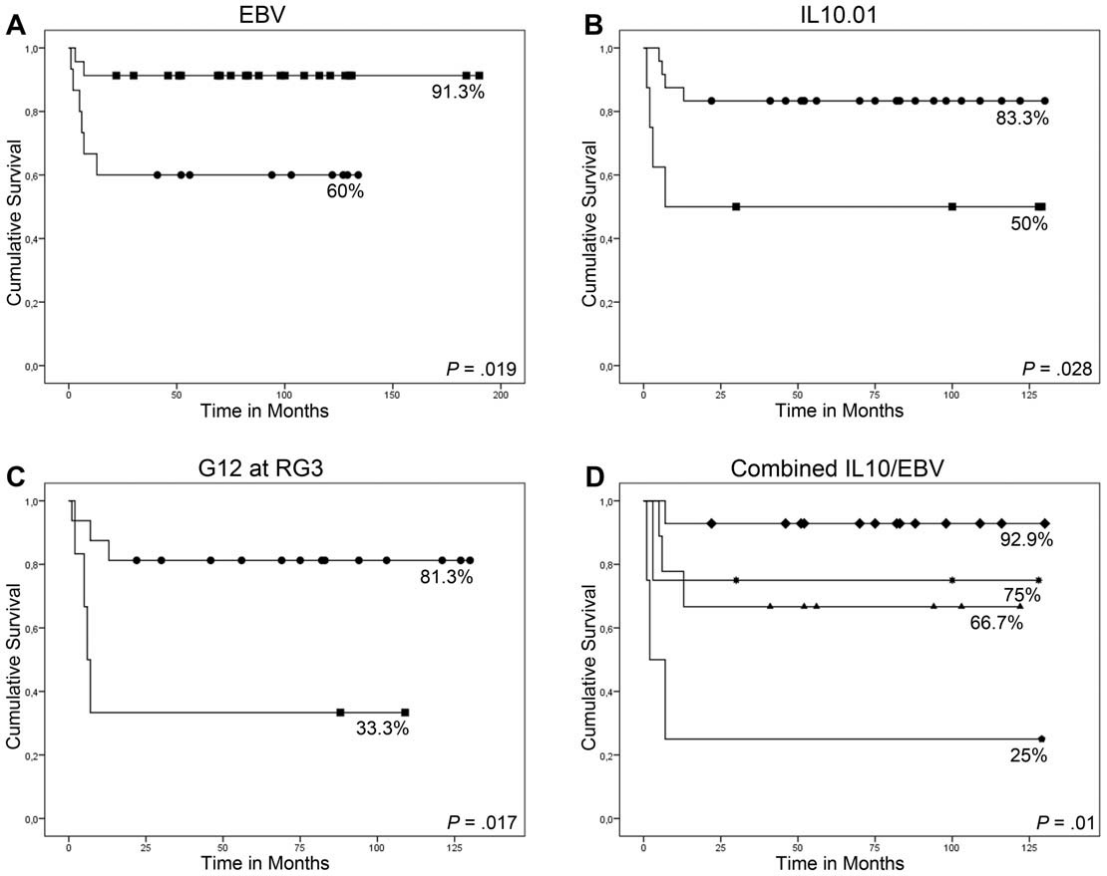
Distributions of Event free survival (EFS) probabilities according to (A) EBV status; (B) presence of IL10.01 family; (C) presence of IL10.G12 microsatellite allele at RG3; ■ positive; • negative; (D) IL10/EBV combination of risks; ▲EBV−/IL10.01−; ✱EBV+/IL10.01+; 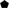
EBV−/IL10.01+; ♦ EBV+/IL10.01−.

EBV status was associated with therapy response, with a better EFS observed in the EBV+ compared to the EBV− patients (91.3% vs. 60%, *P* = 0.019, Fig. 2A). These differences reflected the prognostic value of EBV in the RG3 (EFS 91.7% vs 45.5%, *P* = 0.014; EVB+ and EBV−, respectively) compared to RG2 (90.9 vs. 100%, respectively; *P* = 0.54).

When *IL10* polymorphisms were analyzed in respect of clinical response, the presence of IL10.01 family (R3-GCC) was related to a poor outcome (EFS 50% vs. 83.3%, *P* = 0.028, Fig. 2B). Again, these differences reflected the prognostic value of IL10.01 in RG3 (25% for IL10.01 carriers vs. 75% for non-carriers, *P* = 0.017), compared to RG2 (75% for carriers vs. 100% for non-carriers; *P* = 0.18).

As for the microsatellite IL10.G, when each allele was analyzed separately, allele G12 was related to a poor EFS (55.6% vs. 85.2%, *P* = 0.055) with a significant statistical impact in patients in RG3 (33.3% vs. 81.3%, *P* = 0.017) compared to RG2 (100% vs. 90.0%, *P* = 0.65, Fig. 2C). None of the other *IL10* promoter polymorphisms were associated to outcome. Multivariate Cox analyses confirmed the independent prognostic value of EBV, IL10.01 and IL10.G12 (Table 4).

**Table 4:** Multivariate Cox's proportional hazards regression model showing significant independent predictors of event free survival in Burkitt lymphoma.

Variable	B coefficient	Hazard Ratio	95% CI	*P*
EBV	1.904	6.71	1.254–35.945	0.026
IL10.01 family	−1.59	0.204	0.046–0.9	0.037
G12 allele	−1.55	0.211	1.25–35.94	0.045

When patients were grouped according to the presence of these two prognostic factors, patients EBV+/IL10.01− showed the best EFS (92.9%), while patients EBV−/IL10.01+ showed the worst EFS (25%); intermediate to these groups were the patients with the presence of one of the good prognostic factors, EBV+/IL10+ (75%) or EBV−/IL10.01− (66.7%), (*P* = 0.01). The statistical significance of this model augmented when stratified for RG3 (*P* = 0.001; Fig. 2D). None of the survival comparisons were affected by patients' sex and age.

## Discussion

The role of environmental factors in the pathogenesis of BL has been largely studied, mainly in the endemic form of the disease, in which malaria, age of EBV infection, and nutrition status, among others, have been implicated [9], [29]. EBV is an important environmental factor associated to ∼50% of BL cases in our region, with a proposed role for early EBV primary infection in the pathogenesis of the young BL cases [8]. However, little is known about the genetic constitution of people affected by the disease at individual and population levels. In the present study a higher than expected frequency of *IL10* −1082GG genotype and GCC haplotype was detected in BL patients, associated to an approximately 2.5-fold augmented risk of lymphoma development, suggesting that *IL10* functional variants could contribute to the genetic context in which BL develops. In addition, the analysis of a uniformly treated group of patients showed that the presence of EBV in tumor cells was associated with a favorable outcome, especially in the advanced risk group, while IL10.01 family and IL10.G12 allele were associated to an unfavorable outcome.

Despite the well described interactions between EBV and IL10, in this study we did not observe significantly different frequencies of *IL10* polymorphisms in EBV+ or EBV− groups. Since EBV-associated BL in our region, as well as in the African endemic subtype, affects very young children, it is possible that *IL10* (either human or viral) plays a more complex role in age-specific risks associated to EBV primary infection, which cannot be addressed with the design of the present study.

The prognostic value of EBV in BL has been investigated in a few studies, since a proper approach to this question is prevented mainly because of the low frequency of EBV+ or EBV− cases seen in sporadic or endemic areas, respectively. With a similar number of EBV+ and EBV− cases originated from the same geographical region, we have been able to disclose a favorable effect of EBV on the rate and duration of EFS in our pediatric BL series. A recent work from a similar epidemiologic setting of Brazil (São Paulo State) showed also a better, albeit non significant outcome for EBV+ BL cases [7]. It is likely that the failure to find a significant statistical association of the above mentioned study was due to the inclusion of patients treated with two different protocols, over a long period, along which improvement of support conditions could have influenced survival of the overall group. This was indeed the reason of having selected a uniform group of patients to investigate the prognostic value of EBV and *IL10* genetic variants, since differences in outcomes of BL patients treated with the same protocol before and after 1999 were observed in our institution [24].

At present, we do not have an explanation for the association of EBV with a better therapeutic response. In a group of children diagnosed with classical Hodgkin lymphoma in the same period from the same institution, we have also observed a favorable outcome associated with the presence of EBV in tumor Hodgkin-Reed Sternberg cells [30]. It is possible that in children, where EBV tumor association may be a late complication of EBV primoinfection, clinical behavior might be associated with a differential immune response to viral antigens, as seems to be reflected in the microenvironment composition of pediatric cHL [30]. Conversely, in EBV-associated adult lymphomas, impaired immunosurveillance might be at the root of the pathogenesis and of the unfavorable therapeutic response observed in this clinical setting [31]–[33]. In our patients a particularly favorable response was observed in EBV+ high-risk BL patients. One possible, yet unproven, explanation is that a high tumor burden would be associated with an augmented rate of spontaneous lytic entry in the EBV+ cases, which would potentiate chemotherapy effects. In fact, in endemic BL, where EBV is present in virtually 100% of cases, the expression of the lytic transactivactor Bzlf1 (Zebra) in BL cells was shown to be associated with a favorable response to treatment [34]. At present, promising protocols to treat EBV-associated cancers are based on the induction of EBV lytic or latent expression patterns, aiming to modify phenotypic characteristics and collaborate in the lysis of tumor cells [35]–[37].

To our knowledge, this is the first study to correlate *IL10* genetic variations with susceptibility and therapeutic response in a homogeneous series of pediatric BL. In other B-cell NHL, large studies identified *IL10* polymorphisms as significant risk factors for specific groups of NHL, such as diffuse large B-cell lymphoma [38]–[40]. However, association of specific *IL10* genetic variants with therapeutic response has not been clearly demonstrated [41], [42].

Despite some controversies, most authors agree with an association of −1082G allele in homozygosis with high IL10 expression [15], [16], [43], [44]. Specific microsatellite alleles, such as IL10.R2 and G12 were also shown to be associated with higher IL10 levels [45]. Thus, the role of IL10 in BL pathogenesis might probably be related to the stimulation of B lymphocytes proliferation, coupled with the antiapoptotic properties of this cytokine [13], [46]. Moreover, in BL, the production of IL10 by tumor cells might stimulate intratumoral macrophages to differentiate and to promote B cell survival [47]. Therefore, the main effector of the inter-individual differences of IL10 expression, either B cells or macrophages, is at present undefined.

Regarding therapeutic response, the presence of IL10.01 family and IL10.G12 allele were associated to an unfavorable outcome. The haplotype family (IL10.01) includes the component GCC related to a high IL10 production [17], [43], [44], what was in fact identified as a risk factor in our case-control study. IL10.G12 is among alleles (G10, G12 e G14) also associated to the induction of high levels of IL10 [45].

We are aware of the limitations imposed to our results by the lack of statistical power of our sample number, the need of future studies to replicate our case-control results and the establishment of the mechanisms responsible for the effects of EBV and IL10 on BL clinical response. Besides, we were not able to assert that cases and controls are similar in respect of the genetic background since we have not ethnically matched them. In that regard, the fact that the patients shared the same socio-geographical origin than controls may help mitigate the effect of a potential genetic sub-structure in this study. Moreover, the inter-ethnic admixture of the Brazilian population, among Europeans, Africans and Amerindians, is depicted in genetic analysis as varying over wide ranges in a continuum across the categories of self-reported ethnicity [48], [49]. This view of the genetic variations of Brazilians supports strategy of control selection based on biogeographical ancestry instead of race or color, as claimed for pharmacogenetic studies in Brazil [50].

In sum, with modern intensive chemotherapy regimens, BL is cured in about 80–90% of cases [51]. However, early relapse and refractoriness are an actual limitation of BL treatment, due to the lack of therapeutic options. Identification of biological markers for risk stratification, mainly in the high risk patients, is a current goal in BL research. Our preliminary results indicate that EBV and *IL10* polymorphisms are worth to further investigation in pediatric BL, as they may represent useful clinical biomarkers.

## Supporting Information

Table S1 **Technical information about IL10 genotyping PCR assays.**
(DOC)

Table S2 **Comparisons of genotype, haplotype and family frequencies between children and adult controls.**
(DOC)
