# Supplementary material for: Relationship of Epstein-Barr Virus and Interleukin 10 Promoter Polymorphisms with the Risk and Clinical Outcome of Childhood Burkitt Lymphoma
Source: PLoS One. 2012 Sep 27;7(9):e46005. doi: 10.1371/journal.pone.0046005 (PMC3459931; doi:10.1371/journal.pone.0046005)
Supplement: Table S1 — Technical information about IL10 genotyping PCR assays. (DOC) [file pone.0046005.s001.doc]

| **Table S1**. Technical information about IL10 genotyping PCR assays | | | | |
| --- | --- | --- | --- | --- |
| **Primers** | **Sequence (5´🡪 3´)** | **Function in PCR reaction** | **PCR thermal profile ^1^** | **Expected size of fragments** |
| **-592A/C** |  |  |  |  |
| -592con  -592A  -592C | GTGACGTGGACAAATTGCCCATTC (Forward) ACACATCCTGTGACCCCGCCTGTA (Reverse)  ACACATCCTGTGACCCCGCCTGTC (Reverse) | Consensus  Allele specific A Allele specific C | 95^o^C 60s; 60^o^C 60s; 72^o^C 60s;  35 cycles | 200 pb |
| **-1082G/A – IL10.G** |  |  |  |  |
| JW –F^2^  B1  B2 | AGCAACACTCCTCGTCGCAAC (Forward)  CCTATCCCTACTTCCCCC (Reverse)  CCTATCCCTACTTCCCCT (Reverse) | Consensus  Allele specific G  Allele specific A | 95^o^C 60s; 59^o^C 60s; 72^o^C 60s;  30 cycles | 134 to 150 bp |
| **IL-10.R** |  |  |  |  |
| 10.R-S²  10.R-AS | GTTTCTGTAGAGATAGGAGGTGGTAAAGTCCA  CCCTCCAAAATCTATTTGCATAAG | Forward  Reverse | 95^o^C 60s; 60^o^C 60s; 72^o^C 60s; 32 cycles  60^o^C 45min | 195 to 203 bp |
| **Haplotyping** |  |  |  |  |
| -1082A  -592C  -819T  -1082G  -819C | CTACTAAGGCTTCTTTGGGAA (Forward)  CCAGAGACTGGCTTCCTACAGG (Reverse)  GCAAACTGAGGCACAGAGATA (Reverse)  CTACTAAGGCTTCTTTGGGAG (Forward)  CAAACTGAGGCACAGAGATG (Reverse) | ACC  ATA  GCC | 95^o^C 20s;  65 to 63^o^C 30s; 72^o^C 60s; 2x each;  95^o^C 20s; 62^o^C 30s; 72^o^C 60s; 24 cycles | 490 bp  263 bp  263 bp |
| **Β-actin** |  |  |  |  |
| ACT-S  ACT-AS | TCACCCACACTGTGCCCATCTACGA  CAGCGGAACCGCTCATTGCCAATGG | Forward  Reverse | 95^o^C 60s; 61^o^C 60s; 72^o^C 60s; 35 cycles | 300 bp |
|  |  |  |  |  |

^1^All thermal profiles started with a 5-minute segment, to activate the hot-start *Taq DNA Polymerase* and ended with a 10 minute 72ºC segment. PCR reactions were carried out with a final volume of 30µl, 0.2 mM of each dNTP, 0.2 uM of each primer, 0.5U of Platinum *Taq DNA Polymerase*, (Invitrogen, Carlsbad, CA, USA) and 50 ng of DNA. ^2^ For IL10.G and IL10.R genotyping, JW-F and 10.R-S primers were labeled at 5’ with 6-FAM.
