# Supplementary material for: Relationship of Epstein-Barr Virus and Interleukin 10 Promoter Polymorphisms with the Risk and Clinical Outcome of Childhood Burkitt Lymphoma
Source: PLoS One. 2012 Sep 27;7(9):e46005. doi: 10.1371/journal.pone.0046005 (PMC3459931; doi:10.1371/journal.pone.0046005)
Supplement: Table S2 — Comparisons of genotype, haplotype and family frequencies between children and adult controls. (DOC) [file pone.0046005.s002.doc]

Table S2. Comparisons of genotype, haplotype and family frequencies between children and adult controls

|  | **Children (%)** | **Adults (%)** | ***P^3^*** |
| --- | --- | --- | --- |
| **-1082** | **N=63** | **N=153** | ***0.79*** |
| AA | 32 (50.8) | 70 (45.8) | 0.54 |
| AG | 25 (39.7) | 67 (43.8) |  |
| GG | 6 (9.5) | 16 (10.5) |  |
| **-592** | **N=59** | **N=146** | ***0.17*** |
| CC | 26 (44.1) | 64 (43.8) | 1.00 |
| CA | 30 (50.8) | 62 (42.5) |  |
| AA | 3 (5.1) | 20 (13.7) |  |
| **-1082/-819/-592^1^** | **2N=118** | **2N=292** | ***0.293*** |
| ACC | 48 (40.7) | 95 (32.5) | 0.43 |
| ATA | 36 (30.5) | 102 (35) | 1.00 |
| GCC | 34 (28.8) | 95 (32.5) | 0.44 |
| **IL10 family^2^** | 2N=98/100^4^ | 2N=249/260^4^ | **0.39** |
| 01 (R3-GCC) | 11 (11.0) | 34 (13.1) | 0.69 |
| 02 (R2-ACC) | 41 (41.0) | 81 (31.1) | 0.31 |
| 03 (R2-GCC) | 19 (19.0) | 49 (18.9) | 0.86 |
| 04 (R2-ATA) | 27 (27.0) | 85 (32.7) | 0.86 |

^1^ Numbers represent the number of individuals carrying each IL10 proximal haplotype, from 59 children and 146 adult controls; ^2^ Numbers represent the number of individuals carrying each IL10 family; ^3^*p* values column: for each category, the first p value (*italics*) represents the comparison of BL cases vs. controls, by Fisher’s exact test or χ2 test; and the following represent the significance of the odds ratio (OR), which were calculated by logistic regression, having children vs. adults control as dependent variable and each IL10 haplotype or family as a covariate; ^4^The number of chromosomes analyzed does not match with the number of total chromosomes because rare families were excluded from the analysis.
